# Motion Inference Using Sparse Inertial Sensors, Self-Supervised Learning, and a New Dataset of Unscripted Human Motion

**DOI:** 10.3390/s20216330

**Published:** 2020-11-06

**Authors:** Jack H. Geissinger, Alan T. Asbeck

**Affiliations:** 1 Department of Electrical & Computer Engineering, Virginia Tech, Blacksburg, VA 24061, USA; jackg7@vt.edu; 2 Department of Mechanical Engineering, Virginia Tech, Blacksburg, VA 24061, USA

**Keywords:** motion dataset, kinematics, inertial sensors, self-supervised learning, sparse sensors

## Abstract

In recent years, wearable sensors have become common, with possible applications in biomechanical monitoring, sports and fitness training, rehabilitation, assistive devices, or human-computer interaction. Our goal was to achieve accurate kinematics estimates using a small number of sensors. To accomplish this, we introduced a new dataset (the Virginia Tech Natural Motion Dataset) of full-body human motion capture using XSens MVN Link that contains more than 40 h of unscripted daily life motion in the open world. Using this dataset, we conducted self-supervised machine learning to do kinematics inference: we predicted the complete kinematics of the upper body or full body using a reduced set of sensors (3 or 4 for the upper body, 5 or 6 for the full body). We used several sequence-to-sequence (Seq2Seq) and Transformer models for motion inference. We compared the results using four different machine learning models and four different configurations of sensor placements. Our models produced mean angular errors of 10–15 degrees for both the upper body and full body, as well as worst-case errors of less than 30 degrees. The dataset and our machine learning code are freely available.

## 1. Introduction

Estimates of human motion for monitoring and analysis are useful in many circumstances. In sports and fitness applications, quantitative data on a person’s kinematics can aid in their training more effectively. Monitoring workers’ postures may help them avoid injuries. Rehabilitation may benefit from quantitative data on a patient’s capabilities, enabling therapists to customize training programs and observe progress. In addition, exoskeletons and prosthetics can benefit from a quantitative understanding of human motion, since they must work cooperatively with the body. Each of these applications is becoming more prevalent, resulting in a need for databases of accurate human motion, and methods for using sensors to estimate human kinematics.

The expanding need for accurate human motion analysis has resulted in a number of motion capture datasets using various techniques, such as optical motion capture, cameras, and wearable sensors [1,2,3,4,5,6,7]. Optical motion capture using markers is a widely accepted, popular methodology for ground-truth pose data. A widely used dataset is the CMU motion capture dataset collected with optical motion capture [3]. This dataset was the most abundant motion capture dataset of its time, in terms of timespan and number of motions, and contains data on 144 participants with more than 9 h of data. The KIT motion capture project [5] introduced the largest optical motion capture dataset to date, which also includes object interaction. The dataset contains 55 participants and approximately 11 h of data [8]. KIT is important because it focuses not only on human motion but also on interaction with objects and the environment.

The largest and most expansive motion capture dataset to date is the Archive of Motion Capture as Surface Shapes (AMASS). AMASS is the most recent step towards much larger motion capture datasets for deep learning and computer vision use. It “amasses” many previously mentioned motion capture datasets [1,2,3,5]. Since many of the motion capture procedures differ, it uses surface shapes to combine different marker placements with the SMPL and MoSH algorithms [9,10]. It contains 43.6 h of motion capture data on 450 subjects, making it by far the largest publicly available motion capture dataset. However, it shares the same constraints as the other motion capture datasets: in it, researchers instruct people to perform actions in areas designed and constrained by the researchers.

Another valuable recent dataset is the Human3.6M dataset [4], which uses both optical motion capture and other sensors. This dataset is heavily benchmarked for human motion prediction using deep learning [11,12,13,14,15,16,17].

While optical motion capture remains the gold standard for accuracy, motion capture has also been facilitated by advancements in pose estimation using cameras. The Kinect from Microsoft and OpenPose from CMU, are two popular examples [18,19]. These systems do not require marker sets on the human body and can be used in a wide range of locations. However, the 3D pose accuracy is generally lower when using these systems.

There are also motion capture solutions using inertial sensors, and these have several advantages [20,21]. Critically, they are usable in outdoor, real-world settings and are not limited to indoor, small-volume laboratory environments. This difference is essential because it allows for the collection of unscripted motion while people perform day-to-day activities. For example, inertial measurement units have been used for activity assessment [22], sports training [23], and monitoring neurological disorders [24]. Thus, inertial sensors can be used to create datasets of natural motion in real-world settings, like work environments and the outdoors. For example, Reference [25] collected inertial motion capture data in environments, like parks, where the participants rode bikes and climbed jungle gyms. Notably, [7] used multi-view cameras and XSens inertial sensors to create a non-marker-based motion capture dataset in a larger area than is possible with optical motion capture. However, despite the range of applications that inertial motion capture systems could benefit, the largest open datasets are from Reference [7,26], which total 90 min and 50 min, respectively, according to Reference [26].

A separate area of literature besides datasets of human motion is work on inferring full-body kinematics based on a small number of inertial sensors (i.e., with sparse inertial sensors). Full-body motion capture using inertial measurement units (IMUs) requires placing an IMU on each body segment, which typically requires at least 15 sensors (including the head, hands, and feet). This can be cumbersome and prohibitively complex for most applications. Instead, using a much smaller number of sensors (1–7) and inferring the wearer’s activities or kinematics is a promising and more practical method.

There have been multiple previous approaches to full-body human motion inference based on sparse inertial sensors. Many groups have taken a hybrid approach, combining video data with inertial sensor data [27,28,29,30]. Most recently, in Reference [30], the authors use inertial sensors and a single video camera to find 3D poses. Using convolutional neural networks to detect 2D poses [19], the authors find 3D postures with help from inertial sensor data.

Depth cameras and optical motion capture have been used alongside inertial sensors as well [31,32]. In Reference [31], the authors use a Kinect and six inertial sensors for full-body motion inference. In Reference [32], the authors propose a real-time system that makes use of six sparse inertial sensors and five reflectors for optical motion capture. Other than inertial sensors, human motion inference has also been studied using magnetic sensors [33,34], foot pressure sensors [35], accelerometers [36,37], and inertial-ultrasonic motion sensors [38].

Still other groups have inferred kinematics based solely on a small number of inertial sensors. To accomplish this, both machine learning and optimization algorithms have been used. In Reference [39], the authors use Gaussian Processes to learn mappings between data from four inertial sensors and optical motion capture data. The inertial sensors are placed on the wrists and ankles. In additional experiments, the authors estimate walking postures with even fewer sensors. However, in more recent works, Gaussian Processes have mostly been replaced by neural networks because those are capable of scaling with training data and generalizing to unseen data.

More recently, Reference [40] made use of densely connected neural networks to predict postures using five inertial sensors with several different configurations. Reference [40] used an XSens MVN Link suit, allowing them to test multiple configurations. However, they only have around 2 h of data collected in lab conditions, and only predict a single posture at a time.

In Reference [25], the authors present an offline optimization method by minimizing orientation, acceleration, and anthropometric errors, and jointly optimizing postures over multiple frames using six inertial sensors. One interesting finding from this paper is the necessity of an acceleration term, which significantly benefits posture approximation. They also made use of an anthropometric term to ensure they generate human-like poses. Overall, they generate accurate human motion from a variety of postures. However, their method is computationally expensive and offline.

Most recently, Reference [26] presents an advancement on Reference [25] that uses deep learning instead of offline optimization. They use a two-layer bidirectional Long Short-Term Memory (LSTM) architecture to predict single postures using 20 past and 5 future frames. Their system uses six inertial sensors and runs in real-time. One interesting point is that they use the AMASS dataset to generate synthetic inertial sensor data, which allows them to generate 45 h of training data. However, because they use synthetic data, they have to fine-tune on real inertial sensor data that they collect manually to perform well on their test set.

Despite the previous work in human motion datasets and motion inference, there remain opportunities for improvement in both areas. Previous human motion datasets have been largely scripted, and may contain large amounts of data corresponding to uncommon motions while leaving out other behaviors, such as subconscious habitual motions. Thus, previous datasets may not be sufficient for applications in day-to-day life where people move, and live, in real-world environments. To aid the study of exoskeletons, robotics, and human-computer interaction, there is a need for datasets that contain significant amounts of unscripted, natural human motion. A truly natural motion dataset would not be directed by a researcher, and would not be constrained to a highly limited environment that contains a limited number of objects with which people can interact.

Additionally, there remain opportunities for improvement in motion inference algorithms using sparse inertial sensors. With the field of deep learning rapidly growing, it is useful to understand how well new algorithms perform relative to previous works. Additionally, the relative accuracy of different sensor configurations has not been previously studied very much.

In this paper, we have three primary contributions. First, we present a new dataset of full-body kinematics as regular people continued with their lives and performed their jobs, which we refer to as the Virginia Tech Natural Motion Dataset. Second, we evaluate two deep learning algorithms and their variants for motion inference. Specifically, we conduct motion inference using Sequence-to-Sequence (Seq2Seq) and Transformer models, which have not been previously used for human motion inference, to understand their accuracy. Third, we compare the relative accuracy of motion inference using different sparse sensor configurations, and for the upper body alone versus the whole body.

## 2. Materials and Methods

### 2.1. Overview

We first provide a brief summary of our work, which consists of (1) the collection of the new dataset of natural human motion, and (2) our machine learning algorithms and experiments.

We captured participants’ full-body kinematics by putting them in an XSens MVN Link suit, and then letting them continue about their day with little to no interference in unconstrained real-world environments. We used the XSens MVN Link because inertial motion capture is well-suited to capturing natural motion, and this system is wireless with an on-body data logger. It is not constrained by motion capture volumes as is optical motion capture, it does not suffer from line-of-sight limitations like cameras, and with recent advancements in its Kalman filter design, it is capable of working near magnetic surfaces.

**Figure 1:**
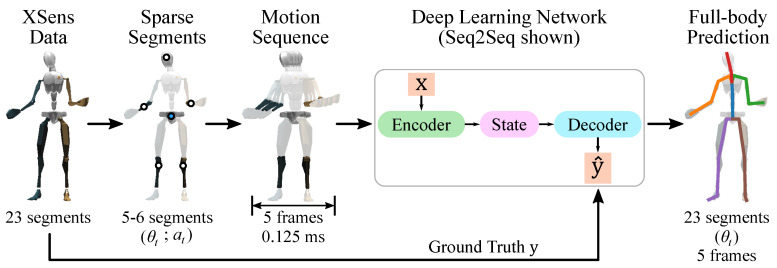
An overview of the approach to full-body human motion inference in this paper. We use a set of sparse segments, pass five frames (0.125 ms) of segment orientation and linear acceleration data into a neural network, and then predict full-body segment orientations for those five frames. The dots on the second mannequin (“Sparse Segments”) indicate the segment locations used to predict the remaining kinematics; the blue dot is the location of the sensor to which the others are normalized. XSens data is used for the input and ground truth. The symbols $\theta_{t}$ and $a_{t}$ refer to orientation and linear acceleration at time *t*, respectively, for each segment. Note that we also study upper-body motion inference (with 15 segments of full body information, and 3–4 segments used as the sparse inputs), and we study Transformers as another deep learning algorithm.

We then use our dataset to conduct human motion inference using sparse segment information. We predict the body’s kinematics using only information from a few segments, with the measured kinematics of all segments as the ground truth. Specifically, we use only 3–6 segment orientations and accelerations as inputs to a machine learning model and we then infer upper-body or full-body segment orientations as the outputs (15 and 23 segment orientations for the upper-body and full-body, respectively). A summary of the setup for deep learning that we study is in Figure 1.

### 2.2. Data Collection

#### 2.2.1. Data Collection System

To collect high-quality kinematics data, we used an XSens MVN Link suit (Xsens North America Inc., Culver City, CA, USA). The XSens MVN Link suit collects synchronized inertial sensor data and post-processes it to construct accurate human kinematics [20,41,42,43]. The data collected and outputted by the XSens MVN Link suit includes measurements for segment position, segment linear velocity, both sensor and segment linear acceleration, both sensor and segment orientation, and segment angular velocity and acceleration.

**Figure 2:**
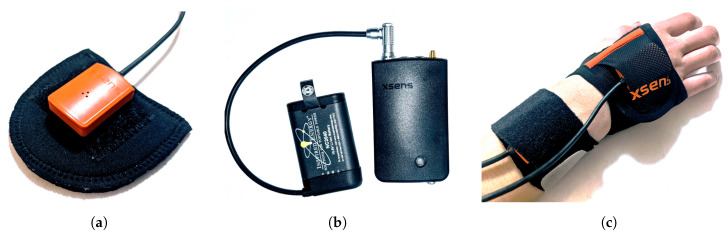
(**a**) An XSens MVN Link inertial sensor (MTX2-4A7G6) placed on a footpad that is inserted into the participant’s shoe under the laces during data collection. (**b**) A bodypack connected to a battery. The bodypack also has two ports for connecting the sensors. The button on the lower right of the bodypack is for turning on/off the bodypack, and for starting a calibration or data collection session. (**c**) A pair of XSens inertial sensors attached to the hand and wrist. The glove contains a pouch with Velcro for attaching an inertial sensor and webbing that constrains the sensor from rotating. The wrist’s inertial sensor was attached to a portion of the strap and then wrapped with the remainder of the strap to constrain it further and avoid any unwanted rotation.

**Figure 3:**
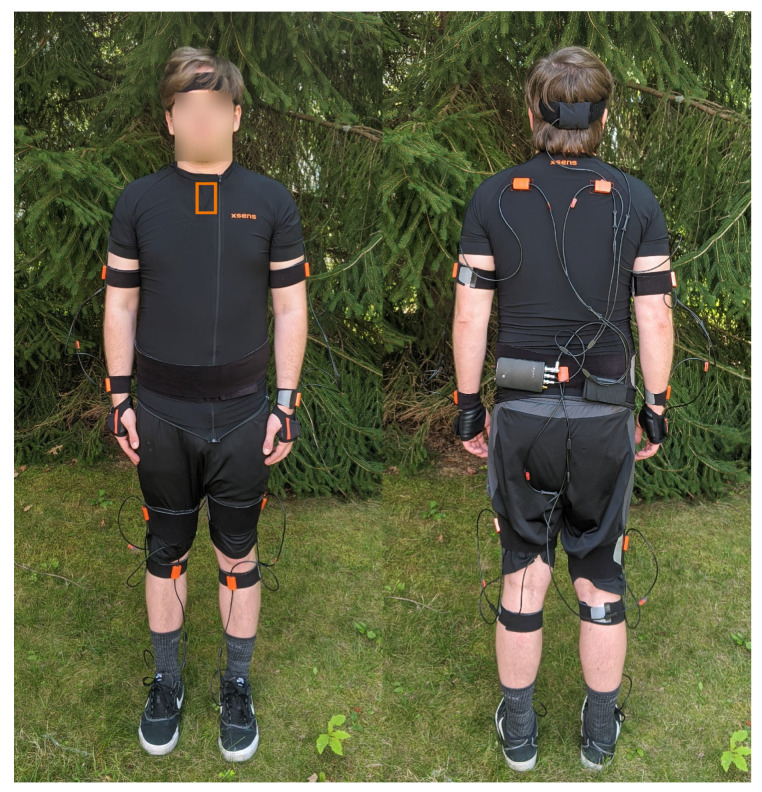
Location and placement of the inertial sensors in this study using the guidelines set by XSens. The sensors were also wrapped with excess webbing, as shown in Figure 2c (not shown in this photo for ease of seeing the sensor locations). Some participants used custom shirts with Velcro patches when the XSens shirt was too small. The wires along the back and limbs were secured and bundled together to make sure they did not catch on objects. Note that there is a sensor on the sternum (indicated by the orange box), but it is inside a pocket on the front of the shirt so is not visible in the photo.

The miniature motion trackers that come with the XSens MVN Link suit have 9 degrees of freedom (DoF) from 3D rate gyroscopes, 3D linear accelerometers, and 3D magnetometers. The motion trackers are placed on different segments of the body to measure the segment’s underlying motion. Figure 2 shows different components of the system, including an individual tracker in Figure 2a, and Figure 3 shows the locations of where the sensors are placed on the body.

The XSens MVN Link consists of 17 inertial motion trackers, along with Velcro straps, gloves, and a headband with tracker pouches, as well as footpads with a Velcro patch to secure a tracker. To log data while people go about their day-to-day activities, an on-body recording device called a bodypack is used for storing data. A battery that has a typical life of 9.5 h powered the bodypack and the sensors. A battery plugged into a bodypack is shown in Figure 2b.

The sensors were Velcroed to segments using a portion of the straps and then wrapped with the remainder of the straps to ensure minimal movement of the sensors. For example, Figure 2c shows the glove with a pouch for a sensor and the wrist with the constrained sensor. This wrapping procedure was followed for each sensor on the arms and legs. The feet and gloves are constrained by the shoelaces and webbing on the glove, respectively, so the wrapping procedure was not necessary for these sensors. Excess wiring was secured to the body to prevent snagging.

The XSens suit records full-body kinematics with mean absolute errors, compared to optical motion capture, of less than approximately $5^{\circ }$ for various tasks, including carrying, pushing, pulling, and complex manual handling [43,44]. This accuracy is important for the creation of a dataset useful for biomechanics studies. XSens uses a specialized Kalman filter design and advanced biomechanical model that compensates for magnetic disturbance, significantly reducing errors due to ferromagnetic materials [41,42]. Thus, materials, like reinforced concrete, that may have been present in surrounding environments would have had minimal impact on the inertial sensor readings. In addition, the motion capture engine allows for data collection under semi-static and highly dynamic conditions. Since our participants were performing daily activities, like lifting boxes, pushing carts, and working at computers, the engine meets our needs.

#### 2.2.2. Data Collection Study Design

Manual material handlers in a home improvement store and students at Virginia Tech were invited to participate. The collection of the manual material handler data and some analysis on the workers’ lifting habits were previously described in Reference [45]. The study was approved by Virginia Tech’s Institutional Review Board (IRB). We collected data from *N* = 17 participants. Each participant was asked if they would be willing to participate in the study and were given at least 24 h to review the consent form before signing it. Measurements were taken of each participant following the guidelines given by XSens. Of the participants, 13 were Virginia Tech students, and 4 were employees of a local home improvement store. Fourteen were male, and 3 were female. The heights of the store employees and Virginia Tech participants were 175.1 cm ± 5.1 cm and 180.7 cm ± 8.1 cm, respectively. The age ranges for the store employees and Virginia Tech participants were 30–58 and 20–35, respectively.

We placed a total of 17 inertial sensors following the guidelines from XSens (Figure 3). Following the wrapping procedure shown in Figure 2c, the inertial sensors were secured to prevent the extraneous movement of the sensors during data collection. The calibration process consisted of the participant going into a neutral pose (N-pose) for 10 s, walking forward and back in a straight line, and then returning to an N-pose. The calibration process serves two purposes. The N-pose allows the XSens MVN Link to perform sensor to segment alignment because the N-pose puts the segments in a known orientation. The walking portion of calibration can help remove magnetic interference from materials, like reinforced concrete.

#### 2.2.3. Manual Material Handlers

The manual material handlers, also known as stockers, worked at a local home improvement store while wearing the XSens inertial sensors. Researchers taught the material handlers (*N* = 4) how to turn the suit on, calibrate the sensors, and turn the suit off. Participants were assisted for 30 min on the first day to answer questions and verify the sensors fit properly. The workers used the suit for the first part of their shift, for up to two hours. Afterwards, they took off the suit to continue their shift.

Workers performed their normal work duties during the sessions, which included opening boxes and containers, lifting and bending to pick up boxes and items, pushing and pulling carts, operating machinery, such as stand-up forklifts, and general motions, such as walking, sitting, and kneeling. The objects they interacted with were on pallets, shelves, and the ground.

#### 2.2.4. Virginia Tech Participants

For the Virginia Tech participants (*N* = 13), data collection was done in the Assistive Robotics Lab located on campus and in the surrounding buildings and stores, as well as one participant’s apartment. The researchers were available on-site throughout the data collection process for troubleshooting any slippage, calibrating the suit, guiding the participants through the calibration procedure, and turning off the system. The participants continued their daily routine on and off the Virginia Tech campus for up to two and a half hours after the start of data collection. For most participants, a calibration file was collected at the start and end of the data collection period to avoid issues with slippage that may have occurred.

The participants from Virginia Tech conducted a myriad of tasks, including performing routine office work, studying, and completing homework while sitting down and standing up; discussing and communicating with other people; walking around campus, getting lunch with people, and going to class; sitting and talking to others in meetings; driving in cars to local stores; collecting data, configuring experiments and orchestrating tests; cleaning and organizing a machine shop and laboratory environment; reading books while lounging in chairs; performing exercises, like Frankensteins, burpees, and pushups; doing spring cleaning by vacuuming and sweeping at their apartment; and other activities of daily life, like napping and playing video games.

#### 2.2.5. Quality Assurance

To ensure data quality, we checked each file using XSens MVN Studio to verify that the calibration was performed correctly. XSens provides a calibration quality estimate, and we flagged and discarded data if the quality estimate was “Poor.” For each file, we cropped and removed any data using XSens MVN Studio where there was evidence that sensors had slipped or fallen off.

### 2.3. Human Motion Inference Using Sparse Sensors

#### 2.3.1. Overview

We studied two scenarios for human motion inference or approximation, whereby the person’s segment orientations are predicted based on a small number of inertial sensors that do not capture the orientations of all of the body segments. We studied upper-body motion inference, where only the kinematics of the upper body were predicted, as well as full-body motion inference. As mentioned before, there are multiple variables provided by the XSens MVN Link suit. The linear segment acceleration and segment orientation are used as a starting point in our study of human motion inference. We use “acceleration” and “orientation” in reference to these measurements in the following discussion. The inputs include the acceleration and orientation data from sparse segments and the outputs include the orientation values (either of the upper-body or the full-body).

**Figure 4:**
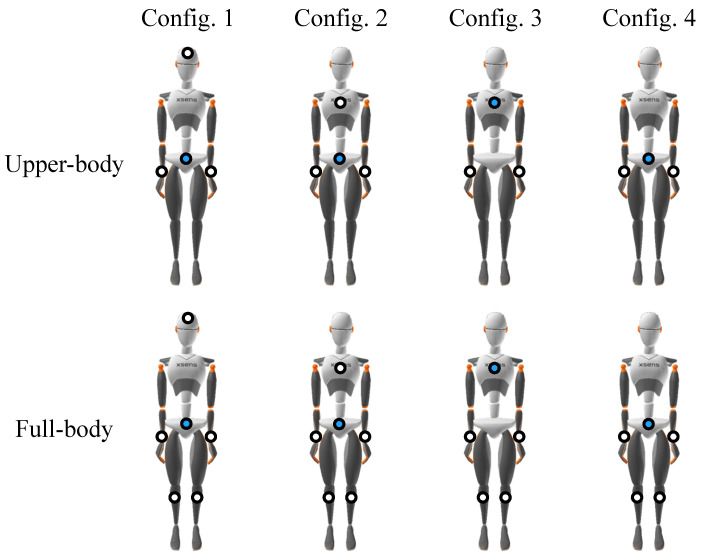
Diagrams of a mannequin showing the inertial sensor locations for the different configurations examined. The white dots denote the segments whose inertial sensor data (segment orientation, acceleration) is passed into the model. These segments are normalized relative to the blue dot, which also has its segment data passed into the model. Note that the only differences between the upper-body and full-body for each configuration are the sensor data about the lower legs. Using this additional information the full-body models are responsible for predicting the orientation of the 8 segments of the lower-body in addition to the upper-body.

We studied how to infer the orientation of the entire upper-body (15 segments, including both arms, the back, the neck, and the head) using sparse segments by evaluating four different configurations (Figure 4). All four configurations use both forearm orientation and acceleration values, which would be provided by inertial sensors worn as wristwatches. Configuration 1 also uses a sensor on the head segment, while Configuration 2 uses a sensor on the sternum segment. The orientation and acceleration of these segments is normalized relative to the pelvis, which contains a fourth sensor. Configurations 3 and 4 only use three sensors total: Configuration 3 uses both forearms normalized relative to a sensor on the sternum, while the fourth configuration uses both forearms normalized relative to a sensor on the pelvis. These configurations are more challenging for kinematics estimation, as they only use three sensors total to infer the kinematics of the entire upper body. In the summary in Figure 4, dots indicate sensor locations, and the blue dot is the location of the sensor to which the others are normalized.

Each of these configurations for estimating the upper-body kinematics require only a small number of sensors, and are plausible for implementation in daily life: wristwatches are commonly worn, and a small sensor could be clipped to a shirt (to sense the sternum) or pants (to sense the pelvis). Upper-body kinematics estimation could be useful for monitoring posture in work environments, approximating the user’s pose in virtual reality applications or other human-computer interaction scenarios, or in studying the entire upper-body during rehabilitation.

We also studied how to infer the orientation of the full-body (23 segments) using sparse segment orientation and acceleration. In total, we study four configurations similar to upper-body motion inference. However, we also use the orientation and acceleration of the lower leg segments (shanks) to help infer lower-body motion, as seen in Figure 4. The orientation and acceleration of the lower legs could be detected with sensors worn on ankle bands, similar to wristwatches.

Our motivation for choosing Configuration 1 for full-body motion inference was to follow the configurations used in previous works that made use of inertial sensors on the wrists, lower legs, head, and pelvis [25,26,30]. We then expanded on this configuration to see if better accuracy was possible using the other configurations. The upper-body motion inference configurations only require the removal of the legs so they are similar to the full-body configurations.

#### 2.3.2. Inputs and Outputs

We frame the upper-body and full-body inference tasks as sequence-to-sequence problems, meaning the inputs and outputs are both sequences over time. For each task, we input a sequence of orientation and acceleration values for a small number of segments and then predict the orientations of additional segments over the same sequence. That is, we predict the segment orientations of the entire body over the same period of time as the input sequence, but the input sequence only includes orientations and accelerations from a few sensors. Our inputs to the models and our ground truth for comparison with the predictions use the data collected from XSens MVN Link system.

The orientation and acceleration values are from each segment following the sensor-to-segment calibration done by the XSens MVN Link. This procedure is done using an N-pose in the calibration process where the segment orientations are assumed to be known [46]. Each segment that is used has an inertial sensor, meaning any end applications could also make use of an N-pose to find the rotation between the sensors’ and segments’ reference frames. In addition, no segment lengths are passed as inputs to make sure the model generalizes between participants.

To construct sequences, we take a sequence from the dataset, which was recorded at 240 Hz, and downsample it to 40 Hz. Downsampling is common practice for human motion datasets. Sampling rates vary from paper to paper so that Reference [4,15,47] use sampling rates of 30 Hz, 25 Hz, and 50 Hz, respectively.

For every task, after downsampling, we have five frames of data as both input and output. This corresponds to a sequence of motion that is 0.125 s long. We explored downsampled inputs and outputs of varying lengths, including: input lengths of five, ten, fifteen, and twenty frames with an output length of five frames; an input length and output length of three frames; and an input length and output length of one frame. After hyperparameter tuning, we found that increasing the input length beyond five frames while keeping the output length constant did not show any measurable benefit. In addition, decreasing both the input and output lengths below five frames resulted in lower accuracy overall. As such, we only report results for the setup with an input sequence length of five frames and an output sequence length of five frames. Longer input lengths also had increased computational complexity compared to an input sequence length of five frames.

For our validation set, we stride over the dataset to test on more data. Starting at index 0, we read *s* frames of data where *s* is the sequence length. Then we move forward by the stride length *l* and read another *s* frames of data. We use a stride of 15 frames in our validation set, whereas, in our training set, we use a stride of 30 frames. This effectively tests on similar sequences from different starting points and “doubles” the amount of data we can test on.

#### 2.3.3. Representing Rotations

Possible rotational representations of the body segments include Euler angles, rotation matrices, exponential mapping, and quaternions. Each rotation representation has benefits, but for Euler angles and rotation matrices, the downsides are too large to ignore. We refer the reader to Reference [48] for additional information on each representation.

Euler angles are a useful representation in $R^{3}$ for visualizing rotations in space, and have a straightforward physical interpretation. However, Euler angles have problems that make them unsuitable for our application, namely singularities and non-uniqueness. Singularities come from gimbal lock where a loss of a degree of freedom can occur when two axes align, effectively “locking” one of the degrees of freedom. Non-uniqueness, which also happens in other representations, like quaternions, occurs because multiple angles on a unit circle can represent the same rotation.

Rotation matrices are another representation that could be used for our application. They avoid gimbal lock and non-uniqueness, but they present a challenge because the set of rotation matrices form the group SO(3) [48]. The columns of a rotation matrix form the basis for a reference frame. Thus, constraining rotation matrices to remain in SO(3) requires the columns to be orthogonal and of unit length. To predict rotation matrices, one must satisfy these constraints by normalizing each column and ensuring orthogonality. In addition to this issue, rotation matrices consist of 9 values, the most of any rotation representation, so they require the most computation. Although these issues are present, they have been used in prior work [26]. However, the authors make no mention of constraining the rotation matrices to SO(3), so it is unclear how they did so.

Another possible representation that avoids the singularities present in Euler angles is the exponential map. Exponential maps, although represented with three dimensions, can avoid singularities through the use of mathematical substitutions and Taylor series (see Reference [48]). The exponential map has been used in prior work in human motion prediction and modeling [11,12,14,47]. Thus, this representation is a reasonable choice in representing rotations, although we did not end up using it.

Quaternions are a 4-dimensional number system that forms a set in $R^{4}$. By constraining them to unit length, we lose a degree of freedom, and we can refer to this new set of quaternions as $S^{3}$. Quaternions have been found to work well in human motion prediction [15], most likely because quaternions have the same local geometry and topology as rotation matrices [48]. However, the constraint that quaternions must have unit length adds extra computational load. This constraint requires a manual normalization layer in the network to enforce unit length so that the predicted rotations are a part of $S^{3}$ [15].

In deciding between exponential maps and quaternions, we decided to use quaternions for several reasons. First, the results from Reference [15] show a benefit in using quaternions that outweighs the normalization layer that must be added to the neural networks. Second, XSens provides segment orientations in quaternion format, which makes them more accessible. Third, we found during testing that quaternions performed better than exponential maps for our use case during pilot testing.

One unaddressed issue with quaternions is that *q* and −q represent the same rotation. We found that the XSens dataset alternates between these representations, resulting in discontinuities. These discontinuities are similar to the findings in Reference [15] that used the Human3.6M dataset for human motion prediction. We made use of their process for enforcing continuity: a function finds where discontinuities in the time series arise and then chooses the representation that maximizes the dot product.

#### 2.3.4. Normalization

To properly learn and generalize, we normalized the orientation and acceleration data that comes as input into the model. This normalization procedure is nearly the same as in other papers such as [26,40]. First, we ensured that the orientation was invariant to the direction the person was facing by normalizing the orientation of each segment relative to that of the root segment. With the root orientation $R_{GP}$, we normalized each segment orientation by:

(1) $$R_{PB}=R_{GP}^{-1}\cdot R_{GB},$$

where R is the orientation of a segment, *G* refers to the global reference frame, *B* refers to the segment’s reference frame, and *P* refers to the root segment’s reference frame. Next, we also normalize the segment’s acceleration data $a_{B}$ relative to the acceleration of the root segment $a_{P}$ and put it in the same frame of reference:

(2) $${\bar{a}}_{B}=R_{GP}^{-1}\cdot \left(a_{B}-a_{P}\right).$$

For configurations 1, 2, and 4, the root segment is the pelvis, while, for configuration 3 the sternum is the root segment.

After this normalization procedure, we also zero the mean and divide by the standard deviation of each feature in the training set. The mean and standard deviation of the training set is used to also normalize the validation set and test set. Since the validation and test data both simulate unseen data collected in the real-world, we are making the assumption that they come from the same underlying distribution as the training data.

### 2.4. Deep Learning for Human Motion Inference

We used two deep learning architectures for motion inference using sparse inertial sensors, sequence-to-sequence (Seq2Seq) and Transformers. We chose to study Seq2Seq models and Transformers because they are effective architectures for learning temporal sequences of data. Since human motion is naturally temporal, Seq2Seq models and Transformers are a good fit for studying human motion as sequences of postures. For example, in the area of human motion prediction (i.e., predicting future kinematics), the Human 3.6M dataset is frequently benchmarked [4], and several papers have used Seq2Seq architectures for motion prediction with great success [14,15]. In comparison, as far as we know, only densely-connected neural networks [40] and recurrent neural networks [26] have been applied to human motion inference. In addition, both Seq2Seq and Transformer architectures have been used in natural language processing, which is another class of sequences of data. In this application, Transformers have come to replace recurrent neural networks and Seq2Seq models. We were interested in determining if Transformers could similarly be used in supplanting recurrent neural networks in processing human motion. In this section, we discuss these models, as well as their training and evaluation.

#### 2.4.1. Seq2seq Architectures

Sequence-to-sequence (Seq2Seq) models were introduced for the task of neural machine translation in Reference [49]. Seq2Seq models consist of an encoder and a decoder, typically containing one or more layers of long-short term memory (LSTM) layers or gated recurrent unit (GRU) layers (Reference [50,51]). The encoder takes in an input of arbitrary length, passes it through the recurrent layers, and produces an encoder hidden state as an output. This encoder hidden state is then fed into the decoder along with a start-of-sequence or <SOS> token.

In Reference [52], the authors introduced bidirectional encoders to Seq2Seq models. Bidirectional recurrent neural networks were originally introduced in Reference [53] as a means to build hidden states by viewing an input sequence in both the forward and backward directions. This approach can improve the encoder hidden state that is shared with the decoder, which then generates sequences. The decoder also contains recurrent layers that output both a hidden state and an output vector. The output vector can then be compared to a target output to compute the loss for the network.

Seq2Seq models were improved following their initial introduction using the attention mechanism in Reference [52], commonly referred to as Bahdanau attention. Attention mechanisms allow for longer input sequences to be evaluated by the decoder by learning which of the encoder hidden states are most important to the sequence being generated by the decoder.

#### 2.4.2. Transformer Architectures

In addition to Seq2Seq architectures, Transformers can be used to study human motion inference [54]. Transformers have emerged as the model architecture of choice in natural language processing because they are capable of modeling natural language effectively across multiple tasks [55,56,57,58,59]. Transformers, similar to Seq2Seq models, have an encoder and decoder, but do not contain recurrent layers. Transformers are of interest to human motion modeling because of the multi-head attention layer present in both the encoder and decoder. Instead of attending to the entire sequence with one attention function as the Seq2Seq model does, the model instead is capable of attending to multiple subspaces of the data at different positions. Since the human body is made up of multiple segments, it is of interest whether Transformers will attend to these segments with multi-head attention and make improvements over Seq2Seq models. Transformers are challenging to discuss in detail; our use of the Transformer is almost identical to the original implementation, so we point the reader to the original paper [54] and two excellent tutorials [60,61].

We make use of the Transformer architecture in two ways. The first is through the use of a bidirectional Transformer encoder similar to Reference [56]. This model is not autoregressive like the Seq2Seq model or the full Transformer, meaning it produces all postures as output at once without predicting one posture at a time. We refer to this architecture as the “Transformer Encoder” in later sections.

The other Transformer architecture we study includes both the encoder and decoder and follows the description in Reference [54]. The model is autoregressive. It will predict one pose at a time, and the decoder can attend to the entirety of the encoder’s outputs as it makes predictions. During training, the entire target sequence is passed into the decoder with a vector of zeros as a start-of-sequence token. The target sequence is masked, so it will only attend to previous target postures. Since the ground-truth target posture is fed into the model during training, the Transformer does not make use of teacher forcing like the Seq2Seq architecture. During inference, the model is passed the start-of-sequence vector, and the first posture is predicted. This posture and the start-of-sequence vector are then passed again to the model. This procedure is repeated until the last posture is predicted.

#### 2.4.3. Training

We experimented with different model architectures using PyTorch [62]. We evaluate four different neural networks for each scenario and configuration. In total, we train 16 different models to compare and contrast the configurations and algorithms. The algorithms are a standard Seq2Seq model, a Seq2Seq model with a bidirectional encoder and attention (referred to as “Seq2Seq (BiRNN, Attn)”), a Transformer Encoder, and a full Transformer.

We conducted hyperparameter tuning using a training and validation set. For each scenario, we used the same training/validation split. We placed Virginia Tech participants 1, 2, 3, 4, 6, 7, 8, 9, 11, and 12 along with workers 1 and 4 in the training set. In the validation set, we placed participant 5 along with workers 2 and 3. We split the data this way to have representative samples from the Virginia Tech participants and manual material handlers in both the training and validation set. Additionally, using different participants for training and validation is important to reflect how these networks could be used in practice, where individuals wearing a sparse set of sensors have their complete kinematics estimated by a pre-trained network. In all, we used 850,114 and 290,888 sequences for training and validation, respectively.

We trained our models on a single V100 GPU. We used the AdamW optimizer [63] with a learning rate of 0.001. For the Transformer models, we multiplied this learning rate by 0.1 every two epochs. We use a batch size of 32 for each model. We found that the models learned quickly using AdamW. We experimented with stochastic gradient descent and found it required around 20 epochs to reach accuracy that AdamW reached in only 3–5 epochs. The models can be trained in under 4 h using a V100 GPU and AdamW.

The loss function used for training is mean absolute error (MAE):

(3) $$MAE=\frac{1}{mn}\sum_{j=1}^{m}\sum_{i=1}^{n}|{\hat{q}}_{i}-q_{i}|,$$

where ${\hat{q}}_{i}$ is the predicted segment quaternion, and $q_{i}$ is the ground truth segment quaternion, and *n* is the number of segments in the body being predicted (15 for the upper-body and 23 for the lower-body), and *m* is the number of frames in the output sequence (5 frames in total).

We tuned the hyperparameters using grid search using the previously mentioned loss function and training and validation sets. For both Seq2Seq models, we found that a hidden size of 512 worked best. For the Transformer Encoder, we used a feedforward size of 200, 2 encoder layers, and *e* heads in the multi-head attention layer where *e* is the dimensionality of the input (4 orientation and 3 acceleration values per segment). For the Transformer, we used two different configurations for upper-body and full-body motion inference. Unlike the other models, we found that different hyperparameter settings worked best for the upper-body and full-body scenarios. We found a feedforward dimension of 2048, two layer encoder and decoders, and four heads in the multi-head attention layers worked the best for upper-body motion inference. We were able to get better results with full-body motion inference when using a feedforward dimension of 512, 6 layer encoders and decoders, and four heads in the multi-head attention layers.

#### 2.4.4. Evaluation

We perform quantitative and qualitative evaluation on two separate test sets that were not part of the training or validation sets. The first test set, which we refer to as the “regular test set,” comes from participants 10 and 13. Participant 10 was on the Virginia Tech campus working on school work in various offices and study lounges. Participant 13 was at their apartment doing spring cleaning (vacuuming, sweeping) and performing other activities, like emptying a dishwasher, playing piano, and playing video games.

The second test set, which we refer to as the “special test set”, contains scripted motions performed by participant 13 that we know for certain are not in the training or validation sets. These motions have much higher accelerations than the training and validation sets and the regular test set. These include physical exercises, such as Frankensteins, burpees, pushups, jumping jacks, and jogging. The test set also includes some low-acceleration motions, as the participant also took a nap while collecting data. We test our models on the special test set to understand how well our models generalize both to kinematic configurations that were not observed in the training set, and to motions with much higher accelerations than those in the training set. As such, the special test set essentially evaluates the worst-case scenario for our models.

We provide the best test results for each model using the mean angle difference $\bar{\theta }$ between the ground truth quaternions *q* and the predicted quaternions $\hat{q}$ for each segment, in degrees:

(4) $$\bar{\theta }=\frac{360}{\pi mn}\sum_{j=1}^{m}\sum_{i=1}^{n}\arccos(|\langle {\hat{q}}_{i},q_{i}\rangle \left|\right),$$

where *i* is indexing the individual body segments, and *j* is indexing the frames in the output, *n* is the number of segments, and 〈·,·〉 is the inner product between two quaternions. We found that MAE (Equation (3)) was more effective as a loss function during training as compared to mean angle difference. There are multiple options for computing the closeness of two quaternions that are less computationally expensive [64]. However, we found that the mean angle difference (Equation (4)) was useful to report differences in degrees.

In addition to quantitative evaluation, forward kinematics was performed after training to validate the models visually and qualitatively. This procedure requires building the kinematic chain starting at the pelvis. The forward kinematics software uses the segment orientations and then multiplies by a single participant’s segment lengths taken from an XSens MVNX file. We used the following equation to perform forward kinematics given the underlying rotation of the segment [46]:

(5) $$p_{G}^{segment}=p_{G}^{origin}+R_{GB}\cdot x^{segment},$$

where $p_{G}^{segment}$ is the position of the target segment’s endpoint, $p_{G}^{origin}$ is the position of the origin, and $x^{segment}$ is the segment’s length. As before, *G* refers to the global reference frame and *B* refers to the segment’s reference frame. As an example, the origin is initially the center of the pelvis. Then using the orientation and the distance from the center of the pelvis to the top of the right upper leg, the position of the top of the right upper leg is determined, which is the right upper leg’s origin. Then using the orientation and the segment length of the right upper leg, the position of the right knee is determined. This is continued throughout all of the the kinematic chains in the body.

Although normalization is performed to improve generalization, we multiply by the orientation of the pelvis to view the posture as it would be viewed without normalization for qualitative evaluation. To do so, we use the following equation on the predicted poses:

(6) $$R_{GB}=R_{GP}\cdot R_{PB}.$$

We present a qualitative evaluation for the full-body scenario as a means of showing what the models are capable of approximating. We provide a set of predictions from a Seq2Seq model, a Transformer encoder, and a full Transformer alongside a ground truth reference posture. All of the postures come from the validation set or the test sets.

## 3. Results and Discussion

In this section, we first describe our dataset, and then provide results on the performance of our algorithms. We describe quantitative results for our algorithms, then show qualitative results to visualize postures predicted by the models.

### 3.1. Dataset

Our dataset of natural human motion contains 40.6 h of data, which is greater than other datasets we know of apart from AMASS [8], which “amasses” data from a multitude of different datasets. Additionally, to our knowledge, it is the only dataset containing natural human motion and only dataset with the kinematics of manual material handlers in a retail environment. Table 1 gives an overview of our dataset in comparison to two other large datasets, and Table A1 in the Appendix A provides additional details. Our dataset includes 35.1 million frames, with 17 subjects of ages 20–58.

Our dataset, the Virginia Tech Natural Motion Dataset, and its metadata are hosted at the Virginia Tech University Libraries website [65]. Our code is also freely available on GitHub (https://github.com/ARLab-VT/VT-Natural-Motion-Processing).

### 3.2. Evaluation Overview

To understand the performance of our models, we conducted studies using quantitative and qualitative evaluations. The quantitative evaluation was in two stages. The first stage covers the performance on the two test sets (regular test set and special test set) using the mean angle difference metric as described in Section 2.4.4. We only run these benchmarks on the two test sets a single time after tuning the model hyperparameters on the separate validation set. We then evaluate each model using histograms of the mean angle difference metric to understand the performance better. For qualitative evaluation, we view various representative postures to determine how well the model generalizes and where it fails. Example postures are taken from both of the test sets and the validation set for qualitative evaluation.

### 3.3. Quantitative Evaluation

We present the model performance with our regular test set for upper-body inference with the four configurations in Table 2, and we present the model performance for full-body inference in Table 3. Overall, the models performed fairly similarly, resulting in mean angular differences of around 10–15${}^{\circ }$ with respect to the ground truth.

Note that the output of the model includes the known segments, mainly for convenience during training and performing forward kinematics. This was also done by other groups in previous work [25,26]. However, the models did not accurately predict these known segments with perfect accuracy. The mean angular differences for all of the known segments combined were between approximately $0.6^{\circ }$ and $1.7^{\circ }$ across the different configurations. Thus, the inclusion of these segments reduces the mean angular difference in this section by approximately $3^{\circ }$. We present the results including these values because the models were trained and optimized using all segments, including the known segments.

Comparing the models to each other, different models perform best in different configurations. For upper-body inference, the Transformer Encoder performs best in Configurations 1 and 4 and nearly is the best in Configuration 3. However, it is around 3${}^{\circ }$ worse than the other models for Configuration 2. Potentially this could be the result of a local minimum during training and validation. The other three models perform nearly as well as the Transformer Encoder, with mean angle differences of $<\;1^{\circ }$ for all configurations.

For the full-body inference, the Transformer model is the best with Configurations 1 and 3, and nearly the best in Configuration 2. However, it is almost 5${}^{\circ }$ worse than the other models for Configuration 4. Again, the other models perform almost as well as the best one, with differences of $<\;1^{\circ }$. The one exception to this is the Transformer Encoder is almost $2^{\circ }$ worse than the others for Configuration 3.

Overall, Configuration 2 has better accuracy than the other configurations for both full-body and upper-body motion inference and all the models. It is interesting comparing Configuration 2 to Configuration 1, which was used in previous works (Reference [25,26,30]); Configuration 2 uses a sensor on the sternum instead of on the head. Configuration 2 does 1.5–3${}^{\circ }$ better than Configuration 1 across all models, suggesting that other studies could improve their accuracy by choosing different sensor locations.

Surprisingly, Configuration 3 provides results for upper-body and full-body motion inference very similar to the results from Configuration 1. Configuration 1 uses sensors on both the pelvis and head, while Configuration 3 instead uses one sensor on the sternum. Thus, Configuration 3 uses one fewer sensor than Configurations 1 and 2, for a total of only three sensors in the upper-body scenario or five for the full-body scenario. In comparison, Configuration 4 also uses one fewer sensor location (but uses the pelvis instead of the sternum) and displays lower accuracy by 2–5${}^{\circ }$ on average as compared to the other configurations. Even with this lower accuracy, the mean angular error is still $<\;16^{\circ }$ for the upper-body and $<\;14^{\circ }$ for the full-body for most models, which is sufficient for many applications.

As mentioned previously, we also conducted a quantitative evaluation using a special test set containing data from participant 13 who performed physical exercises, such as napping, Frankensteins, burpees, pushups, jumping jacks, and jogging. We present model performance on the special test set for upper-body inference in Table 4, and the results for full-body inference in Table 5.

As compared to the regular test set, the special test set has accuracies that are 7–10${}^{\circ }$ worse for the upper-body, and ∼10${}^{\circ }$ worse for the full-body scenario. It is expected that the special test set would have worse accuracies, as the special test set contains unseen motions that we were certain were not in our training data, but these values are promising. Adding dynamic motions to the dataset would likely improve these values.

As with the regular test set, Configuration 2 results in the best accuracy. Configuration 3 again has accuracies several degrees worse than Configuration 2, and Configuration 4 is even worse, although by smaller amounts than with the regular test set.

With the upper-body inference, the Seq2Seq model with the bidirectional encoder and attention performs about as well as the Transformer across all configurations. With full-body inference, the Seq2Seq model with the bidirectional encoder and attention seems to perform best overall. The Transformer does particularly poorly in Configuration 4 for the full-body, around 5${}^{\circ }$ worse than the other models.

**Figure 5:**
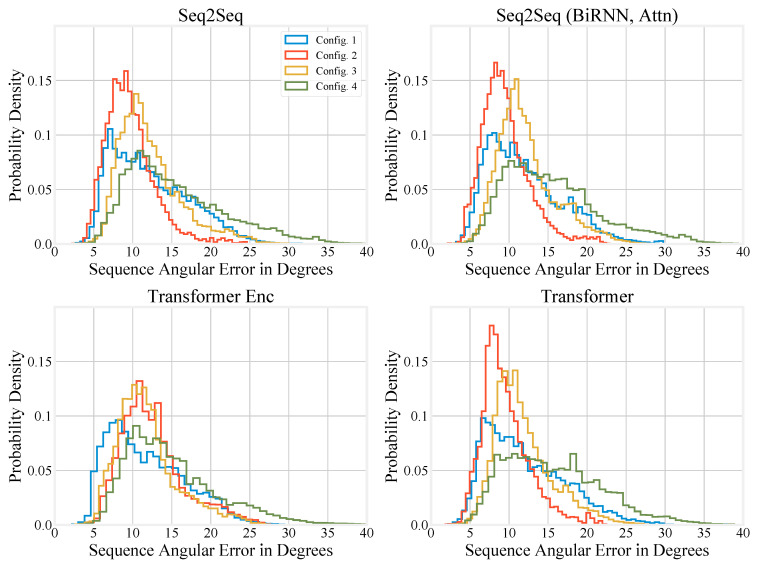
Histograms showing the performance of the different models on the regular test set under different upper-body configurations.

**Figure 6:**
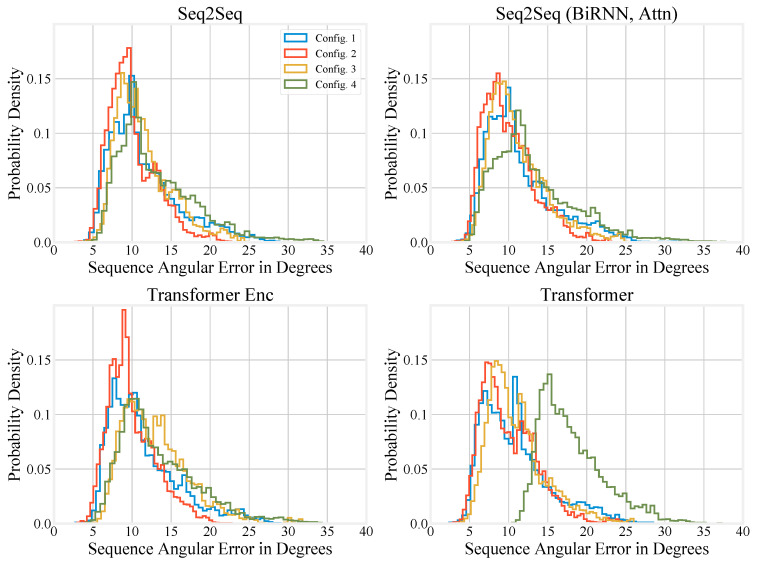
Histograms showing the performance of the different models on the regular test set under different full-body configurations.

Compressing the performance of different models to a single number can be misleading, so we also report histograms of sequence angular error in degrees for upper-body motion inference in Figure 5 and full-body motion inference in Figure 6. We also generated histograms using the special test set, and these are available in the Appendix B.

For the upper-body scenario, it is noticeable that for each model, except the Transformer Encoder, the end of the distribution’s tail drops off faster when using Configuration 2 as compared to the other configurations. Configuration 4 has the worst performance for each model and the longest tail in each case. It is also evident from the histograms that Configuration 3 performs better than Configuration 4, again supporting the conclusion that the sternum is more informative as a root segment for upper-body motion inference than the pelvis.

For full-body inference, the end of the tail in Configuration 2 again drops off faster compared to other configurations, although the effect is not as pronounced as with the upper-body. The center of the distributions for Configuration 2 is also lower, in agreement with Table 3. Overall, the different configurations have histograms that are much more similar than they are with the upper-body inference. However, the Configuration 4 performs noticeably worse when using the Transformer (Figure 6d). Configurations 3 and 4 use the same number of sensors, but Configuration 3 has a shorter tail for most models except the Transformer Encoder.

For both the upper-body and full-body scenarios, the histogram tails have maximum values of around 23–25${}^{\circ }$ for Configuration 2, and 25–28${}^{\circ }$ for Configuration 3. Even with our special test set, the histogram tails have maximum values of around 22–29${}^{\circ }$ for Configuration 2, and 25–30${}^{\circ }$ for Configuration 3. Therefore, our algorithms’ performance is very promising, as the inferred kinematics are not grossly different from the true kinematics, even if they are not perfect.

### 3.4. Qualitative Evaluation

Though quantitative evaluation is useful for viewing concise metrics about the models’ performance, qualitative evaluation is necessary to build intuition for how the models make predictions and where they fail. Our goal in this section is to visualize the postures that the models predict to build intuition about what they get wrong. Qualitative evaluation is performed in almost every study of human motion inference, such as in Reference [26,27,35]. We use Configuration 2 from the full-body scenario to investigate how the models differ. Configuration 2 was chosen because it gave the best results out of the four configurations. The upper-body scenario has very similar outputs, so we only present the full-body results to avoid repetition.

**Figure 7:**
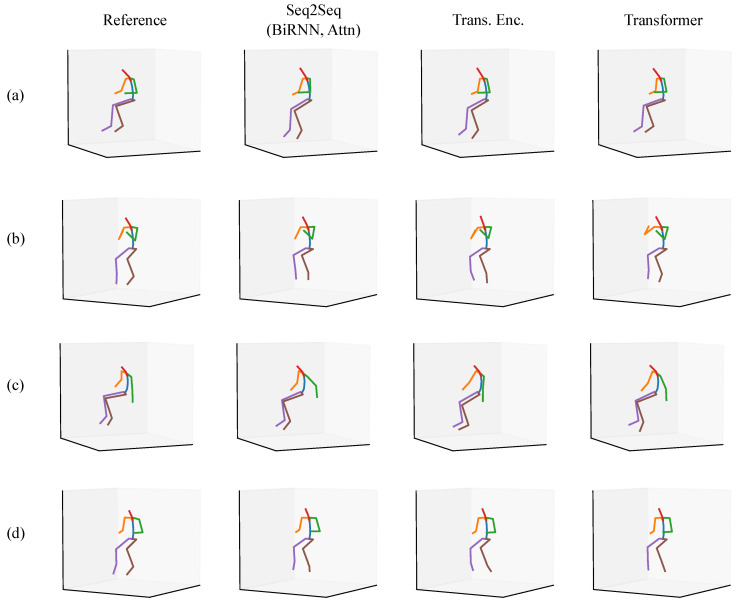
A set of sitting postures from the validation set where the participant went to a drawing/sketching club meeting. The rows are of different postures, such as (**a**) sitting at a desk and typing on a computer, (**b**) sitting at a desk with elbows on the table, (**c**) sitting at a desk reaching down into their bag, and (**d**) sitting at a desk and sketching.

**Figure 8:**
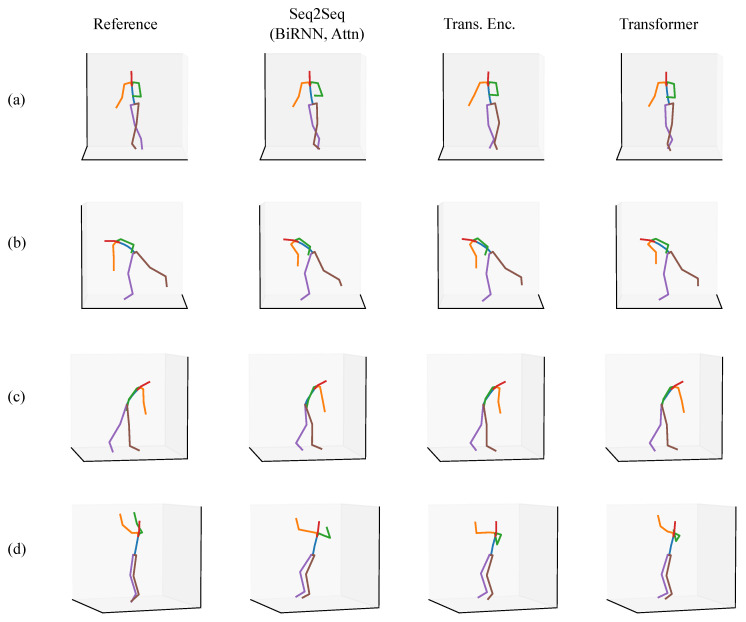
A set of postures from the validation set where the participant was working at a home improvement store. The rows are of different postures, such as (**a**) standing with legs crossed talking with someone, (**b**) reaching for something in a one-legged (“golfer’s”) lift, (**c**) reaching for something with legs split fore-aft, and (**d**) reaching up for something overhead.

**Figure 9:**
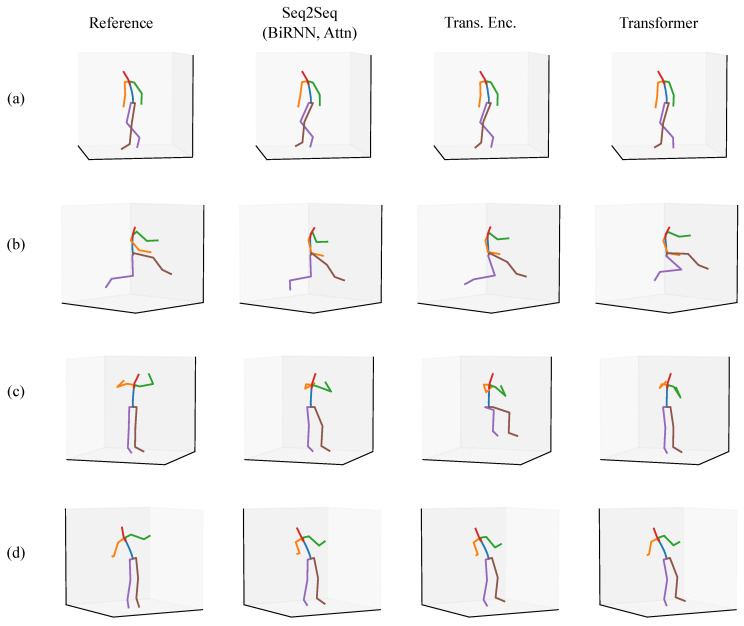
A set of postures from the validation set where the participant was working at a home improvement store. The rows are of different postures, such as (**a**) walking with their hand on a cart, (**b**) kneeling on the ground while reaching for something, (**c**) putting on a vest, and (**d**) lifting something with both hands.

**Figure 10:**
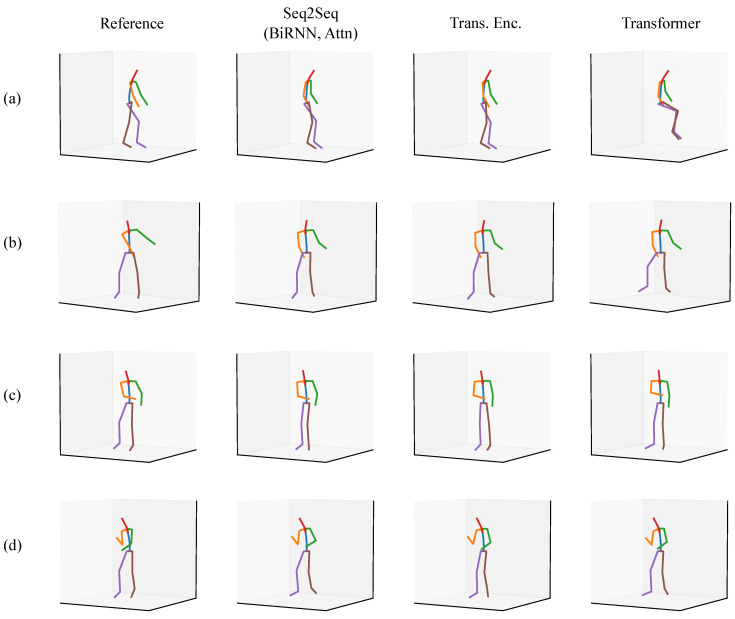
A set of postures from the validation set where the participant was working at a home improvement store. The Transformer had low accuracy (>20${}^{\circ }$) for each of these postures. The rows are of different postures, such as (**a**) operating machinery (possible a stand-up forklift), (**b**) reaching for an object or box across their chest, (**c**) reaching for an object with one hand across their chest, and (**d**) performing some action with high elbow flexion.

**Figure 11:**
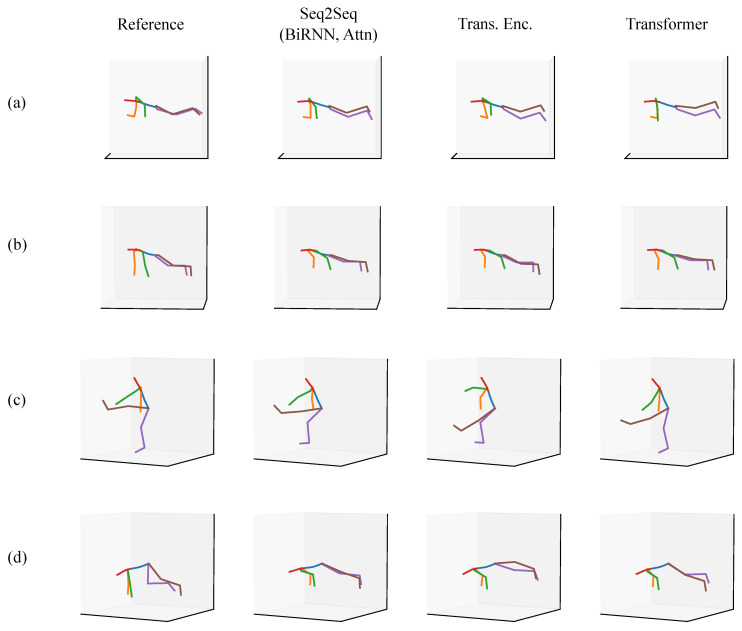
A set of postures from the special test set where the participant was at their apartment. The rows are of different postures, such as (**a**) lying in bed napping ($23^{\circ }$ mean angle difference with Transformer), (**b**) doing push ups ($22^{\circ }$ mean angle difference with Transformer), (**c**) doing Frankensteins ($14^{\circ }$ mean angle difference with Transformer), and (**d**) doing mountain climbers ($23^{\circ }$ mean angle difference with Transformer).

**Figure 12:**
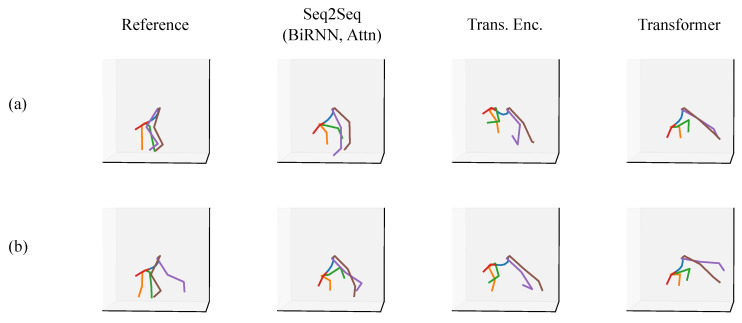
A set of postures from the special test set where the participant was at their apartment. The rows are of different postures during a burpee exercise where the participant does a push up, brings their feet to their hands, and then springs up. The errors for these postures are 61° and 55°, respectively. In (**a**), the participant is bent over fully after bringing their feet to their hands. In (**b**), the participant is moving their feet back to drop into a push up position.

In each of the figures in this section (Figure 7, Figure 8, Figure 9, Figure 10, Figure 11 and Figure 12), we label the different rows with letters (a, b, c, d), representing different postures that the model must infer using sparse segment orientation and acceleration. Our goal was to choose various representative postures, such as standing, sitting, and bending, to understand where the models fail and succeed. The first column is the reference (ground truth) posture, and the remaining columns are predictions from each model. We provide descriptions of the postures in the captions.

The first set of postures comes from participant 5 in our validation set (Figure 7). Of note is that each model correctly predicts the participant is sitting down in each posture. In posture (a), the participant is sitting and typing on a laptop in their lap. Each model has subtle inaccuracies in inferring this motion, but most seem to infer realistic motion. In posture (b), the participant has their elbows on the table, but the Transformer incorrectly infers the participant is lifting their right elbow, and the Transformer Encoder infers inaccurate orientations of their feet. In posture (c), the participant is reaching down into their bag. The Seq2Seq model infers that the person is reaching further back than they are in reality. In posture (d), the participant is sketching instead of typing, and the Transformer Encoder and Transformer both infer inaccurate foot orientation.

Another set of postures comes from worker three, who was working at a home improvement store (Figure 8). Similar to Figure 7, the models can all determine that the participant is standing up instead of sitting down. In posture (a), the participant has their legs crossed while leaning on something. Each model except for the Transformer Encoder accurately models the participant crossing their legs. In postures (b) and (c), the participant is doing a one-legged lift and split-legged lift with a heel raised, respectively. Each model can infer this correctly, though there is some inaccuracy in the right arm orientation for posture (b). In posture (d), the Seq2Seq model and Transformer Encoder fail to infer accurate upper arm kinematics for both arms. The Transformer is reasonably accurate in inferring the right arm’s orientation but is less accurate for the left arm. Each model also fails to infer that the participant is on their toes.

The third set of postures is from worker two, who was also working at a home improvement store (Figure 9). This set of postures is interesting because it demonstrates the range of motions the models can perform inference for, and where the models fail. In posture (a), the participant is walking with their hand on a cart behind them. Each model has accurate inference about the posture. In posture (b), the participant is kneeling on the ground while reaching for something. Each model has varying predictions for how extreme the knees’ flexion is, but each seems to correctly infer that the participant is kneeling. In posture (c), the participant is putting on a vest or jacket. This posture is particularly challenging because of the unusual way the arms move during the activity. The Transformer Encoder, in particular, incorrectly predicts the participant is sitting down. In addition, each model inaccurately infers the upper arm orientation. Each model has varying inaccuracies. Finally, in posture (d), the participant is lifting something with both hands. There are varying degrees of inaccuracy between each model. Each model seems to be conservative about how far apart the arms are.

We also present a typical set of cases where the mean angular error is greater than $20^{\circ }$ when using the Transformer, in Figure 10. These inaccurate predictions are very few in number in our test set and validation set. In Figure 10a, the Transformer predicts that the person is sitting down while they are instead operating machinery (a stand-up forklift). The accelerations from the forklift possibly cause this error to occur. In (b), the model correctly predicts the participant is reaching across their chest, but incorrectly predicts the amount of knee flexion and hip rotation present in the posture. In (c), the participant is reaching across their chest with one hand. The Transformer model output looks reasonable, but this still resulted in a high angular error, likely due to errors in the arms and hip rotation. Finally, in (d), the participant is performing an action with high elbow flexion. The Transformer correctly predicts this but has a large amount of error in predicting the leg orientation.

Our special test set contains further examples of where the mean angular error is high. The first set of cases relates to the participant napping, doing push ups, doing Frankensteins, and doing mountain climbers, shown in Figure 11. In addition, we share two of the worst failure cases from our special test set. These postures are from the participant performing burpees (Figure 12).

The special test set pushed the models to their limits in interesting ways. Because there were no people who exercised in our dataset, the models had lower accuracy when handling these unseen cases. Data of people lying down and napping also was not present in our training dataset because we mainly collected data in workplaces. However, considering the complete lack of data for Frankensteins, pushups, and lying down in our training set, the models generalize reasonably well, and the outputs are reasonable for these exercises. Burpees, on the other hand, are clearly at the limit for the models.

### 3.5. Comparison to Prior Works

Our results compare favorably to other previous work in this area. Other approaches to full-body motion inference, such as Reference [26,40], also make use of neural networks for human motion inference using sparse sensors. In Reference [26], the authors predict Skinned Multi-Person Linear model (SMPL, [9]) parameters of a single frame using 20 past frames and five future frames at test time with a bidirectional Long Short-Term Memory network (LSTM). They synthesize their IMU data using AMASS [8] and then fine-tune on a real IMU dataset that is 90 min in length. An important point is that their system was used in a real-time demonstration; our models can also be run in real-time, but we did not develop a demonstration. Overall, their real-time system and use of SMPL model parameters are impressive. Like our system, theirs has lower accuracy with dynamic and unusual motions.

In contrast, we predict segment orientations instead of SMPL model parameters. We also frame the problem as a sequence-to-sequence problem and use Seq2Seq models and Transformers instead of only LSTMs that predict a single frame of poses. We do not use future frames; we map a sequence of sparse segment orientation and acceleration data to a concurrent sequence of full-body or upper-body orientations.

In comparison to Reference [26], we found that our histograms have shorter tails, which is worth noting as this may lead to fewer failure cases in endpoint applications. Their histogram tails extend past 50${}^{\circ }$, whereas ours are less than 30${}^{\circ }$ even for the special test set. However, this may be due to different underlying motions in each of our datasets and that they use joint angles instead of segment orientations to measure angular differences. Finally, our dataset for training and validation is, to the best of our knowledge, the largest real inertial motion capture dataset and is not synthetic, like the synthetic IMU data in Reference [26]. Our view is that our dataset will be complimentary to other large datasets, such as AMASS and KIT, and may also provide for novel approaches to problems because we collected more data per participant on average in real-world environments.

### 3.6. Limitations and Future Work

Although our natural motion dataset contains around 40 h of human motion in unconstrained real-world environments, the number of participants (*N* = 17) is still limited compared to other motion capture datasets [5,8]. We would also like to make the dataset evenly split between males and females in the future, and this is currently not the case. It is unknown how many unique postures are in the dataset, and manual labeling by humans will be necessary. In future work, this dataset can be expanded upon and labeled.

Another limitation is that the XSens suit only records kinematics. Force sensors could add information about how people are interacting with their environment. FootSee remains a fascinating case study into how force sensors can be used to infer human motion [35]. Future work could incorporate force sensors in people’s shoes to incorporate additional information and expand use cases. In addition, synchronized on-body or off-body cameras could be used to capture additional context about the environment or improve human motion inference accuracy. Other approaches have used off-body cameras and inertial sensors to allow for hybrid motion capture [27,28,29,30]. One exciting approach, called EgoCap, uses two on-body fish-eye cameras for full-body motion capture [66], and a similar approach could be used in conjunction with inertial sensors.

Finally, our dataset was limited in the activities it captured. While the dataset contains 40 h of unscripted, natural human motion that was captured entirely in real-world environments, it did not fully capture all possible activities of daily living, and did not capture many dynamic motions. Data in future training sets should capture activities of daily living more comprehensively and account for more extreme motions from sports or exercising.

An exciting possible direction for future motion capture studies is to make them “occupation”-focused. Previous large-scale datasets have collected data from people in enclosed laboratory spaces where participants perform defined actions. We believe the future of large-scale motion capture projects should be in real-world environments with workers, such as manual material handlers, nurses, factory workers, farmers, and firefighters. Our natural motion dataset is an attempt at collecting natural motion in real-world environments, and we aim to use it to design and study tools and assistive devices that can help people.

## 4. Conclusions

In summary, we introduced the largest full-body inertial motion capture dataset as far as we know, and our dataset is available for both biomechanics and machine learning studies. We also explored Seq2Seq and Transformer models for inferring upper-body and full-body kinematics using different configurations of sparse sensors. Our models produce mean angular errors of 10–15 degrees for both the upper body and full body, and worst-case errors of less than 30 degrees. Overall, our approach leads to reasonable kinematics estimates for typical activities of daily living.

Our study into human motion inference has shown that a wide range of human motion can be inferred effectively with limited segment information using Seq2Seq models and Transformers. Importantly, we found that the sternum is a useful segment for upper-body and full-body motion inference: as a general segment, it provides more information than the head, and, as a root segment, it provides more information than the pelvis. The use of the sternum as a root segment also allows us to perform accurate human motion inference with one fewer segment in full-body and upper-body motion inference. This is a novel contribution of our work, because to the best of our knowledge, other research has not shown or tried to show a direct benefit to using the sternum as a root segment. This has important implications for future applications. This also may have benefits for the practical implementation of a small sensor set. An inertial sensor may not move very much if attached to a person’s glasses or hat, but requiring someone to wear something on their head may be more intrusive than a sensor clipped to the front or back of their shirt.

We also did an extensive study on upper-body motion inference, while other works have focused only on the entire body (an exception is Reference [36]). Using just the upper-body resulted in similar accuracies to the full-body. This is an important contribution as we think many use cases do not require full-body motion to be useful. For example, in stroke rehabilitation exercises where the patient is seated at a table, upper-body kinematics could be inferred with only two inertial sensors on the patient’s wrists and one clipped to the back of their shirt. Such an application could help remediate privacy concerns over the use of cameras for motion capture while giving constructive feedback about the patient’s posture and motion.

We found that some postures are hard to model with sparse segment information, such as putting on a jacket or reaching for a high object, and these postures led to larger errors. However, even in these cases, our models tend to predict postures that are plausible. In other words, when viewing failure cases in our validation and regular test set, we did not see cases where the limbs were tangled together, or unrealistic joint angles were predicted. Thus, these models are promising for real-world applications and additional use with future datasets.

## Figures and Tables

**Table 1:** An overview of the natural motion dataset presented in this paper, and comparison to the largest comparable datasets. Further information about the dataset is in the Appendix A in Table A1. Our dataset has a comparable number of hours to two other large motion capture datasets, Archive of Motion Capture as Surface Shapes (AMASS) and KIT, although they have many more subjects than our dataset. Our dataset included more hours per subject since subjects did everyday activities in real world environments outside of laboratories. A benefit of KIT is that it includes object interaction, hand motions, and other data on top of full-body kinematics. In the table, MoCap stands for Motion Capture, and MoSh++ is the improved version of Motion and Shape capture [10].

	Ours	AMASS [8]	KIT [5]
Hours of Data	40.6	45.2	37.2
Number of Subjects	17	460	224
M/F	13/4	N/A	106/37
Avg. Hours/Subject	2.39	0.10	0.17
Lab Environment	No	Yes	Yes
Body Kinematics Only	Yes	Yes	No
Methodology	Inertial MoCap	SMPL [9], MoSh++	Optical MoCap

**Table 2:** The performance of the various models with differing configurations for upper-body motion inference with the regular test set. The values reported are the mean angle difference in degrees for the test set. Each configuration uses the entire upper-body as output (15 segment orientations with the pelvis included). Figure 4 shows diagrams of the configurations. Bolded values are the minimum in each column, and bolded gray values are within $0.5^{\circ }$ of the minimum.

Model	Config. 1	Config. 2	Config. 3	Config. 4
Seq2Seq	**12.29**	**9.64**	**12.26**	15.64
Seq2Seq (BiRNN, Attn)	**11.99**	**9.55**	**12.30**	15.69
Transformer Enc.	**11.82**	12.56	**11.88**	**15.08**
Transformer	**12.17**	**9.58**	**11.87**	15.77

**Table 3:** The performance of the various models with differing configurations for full-body motion inference with the regular test set. The values reported are the mean angle difference in degrees for the test set. Each configuration uses the full-body as output. Figure 4 shows diagrams of the configurations. Bolded values are the minimum in each column, and bolded gray values are within $0.5^{\circ }$ of the minimum.

Model	Config. 1	Config. 2	Config. 3	Config. 4
Seq2Seq	11.54	**10.00**	**11.44**	**13.25**
Seq2Seq (BiRNN, Attn)	11.48	**10.05**	**11.16**	**13.34**
Transformer Enc.	11.53	**9.98**	12.97	**13.45**
Transformer	**10.94**	**10.06**	**11.00**	18.16

**Table 4:** The performance of the various models doing upper-body motion inference with the special test set. The values reported are the mean angle difference in degrees. Each configuration uses the entire upper-body as output (15 segment orientations with the pelvis included). Figure 4 shows diagrams of the configurations. Bolded values are the minimum in each column, and bolded gray values are within $0.5^{\circ }$ of the minimum.

Model	Config. 1	Config. 2	Config. 3	Config. 4
Seq2Seq	19.38	18.52	22.18	22.85
Seq2Seq (BiRNN, Attn)	**18.49**	**17.40**	**20.23**	22.83
Transformer Enc.	**18.12**	19.95	22.92	22.52
Transformer	18.71	**17.98**	**19.96**	**21.02**

**Table 5:** The performance of the various models doing full-body motion inference with the special test set. The values reported are the mean angle difference in degrees. Figure 4 shows diagrams of the different configurations. Bolded values are the minimum in each column; there are no values within $0.5^{\circ }$ of the minimum in this table.

Model	Config. 1	Config. 2	Config. 3	Config. 4
Seq2Seq	21.35	22.03	**20.65**	23.17
Seq2Seq (BiRNN, Attn)	**20.58**	**19.86**	21.48	24.51
Transformer Enc.	22.54	20.78	23.36	**22.83**
Transformer	21.64	22.30	22.08	28.12

**Figure A1:**
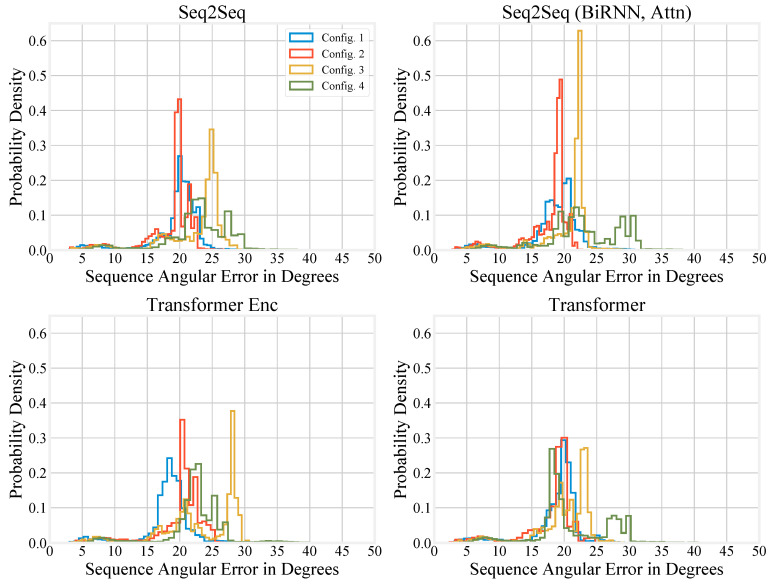
Histograms showing the performance of the different models on the special test set under different upper-body configurations.

**Figure A2:**
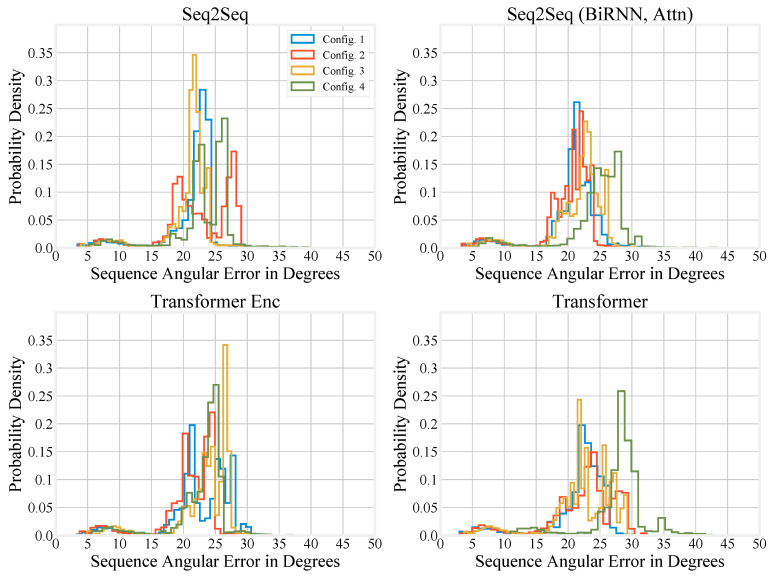
Histograms showing the performance of the different models on the special test set under different full-body configurations.

## Appendix A. Dataset Description

**Table A1:** Metadata for our dataset. Subjects (“Subj.”) with a “P” prefix were participants on or near Virginia Tech’s campus, while a “W” prefix indicates participants at a home improvement store. The number of frames in the log (“# Frames”) and the duration (“Dur.”) in hours are specified. The Notes column describes the general activities the participants were doing during data collection.

Subj.	Day	# Frames	Dur.	Notes
**All**	**All**	**35087845**	**40.59**	
P1	Day 1	1557894	1.8	The participant was in a lab environment operating an experiment involving a virtual reality setup and other hardware. They stand and sit repeatedly to make adjustments. They manipulated many things with their hands.
P2	Day 1	1103415	1.27	The participant was sitting in a chair in an office environment, performing work on a computer that included CAD design. The participant was also talking with others and walking around. The participant was having a technical discussion with another person throughout the duration of the data collection.
	Day 2	1293893	1.5	The participant was working in a lab environment, manipulating scrap metal and other materials with their hands. They also operated a drill press. The participant was talking with other people, working with his hands, and walking around an office.
	Day 3	1604016	1.85	The participant was working in a lab environment, manipulating scrap metal and other materials with their hands. They also operated a drill press. The participant was talking with other people, working with his hands, and walking around an office.
	Day 4	1349583	1.56	The participant was cleaning up a lab environment and machine shop. They stand and walk around for most of the trial while manipulating objects with their hands.
P3	Day 1	1377289	1.6	The participant was in an open office/lab environment at their desk. The participant was reading and writing on a tablet while sitting and reclining in their chair. The participant also walked around some, spoke with others, and manipulated things with their hands.
P4	Day 1	1709725	1.97	The participant was sitting in an office environment working at a computer. The participant was sitting and manipulating things at their desk for the majority of the trial.
	Day 2	529900	0.61	The participant was sitting at a desk in a lab environment doing office work.
P5	Day 1	272731	0.32	The participant attended a sketching session. They walked to the sketching session and drew for the remainder of the log. They did other interesting things, like hold the door open for people, reach into their backpack while sitting down, and operate their laptop in their lap.
P6	Day 1	1709741	1.98	The participant was sitting at a desk, working on a computer, and talking with others.
	Day 2	1242864	1.44	The participant was standing for the duration of the log and operating an experiment. They were primarily interacting and talking with an experiment collaborator and the participant who was testing an exoskeleton and assisting with data collection. They were leading a person through the experiment, handing them weights, standing, and performing other actions.
P7	Day 1	1256115	1.45	The participant walked around Virginia Tech’s campus, went to class, and also drove around in their car to a local department store.
P8	Day 1	1894520	2.19	The participant walked around campus, attended office hours, and worked on homework with their computer while sitting down.
P9	Day 1	3299680	3.82	The participant walked across campus to meet a friend for lunch, sat down, ate food, and did stretching motions to show their friend how the XSens worked. They also went to class, took notes, and spoke with people about the XSens suit.
P10	Day 1	1358481	1.57	The participant walked around campus to a teacher’s office hours and worked in the office. They worked on a computer and on their homework while standing up.
P11	Day 1	2574733	2.98	The participant walked across Virginia Tech’s campus to attend class. The participant then walked back across campus, got take-out food from a restaurant, and returned to the starting location.
P12	Day 1	1709704	1.98	The participant walked across campus to a lab meeting and their office where they sat and discussed things with other people.
P13	Day 1	1587648	1.84	The participant was at their home near Virginia Tech’s campus. They were doing Spring cleaning activities, such as sweeping and vacuuming. They also emptied a dishwasher, played the piano, played video games, and took a nap.
	Day 2	209547	0.24	The participant did exercises at their home near Virginia Tech’s campus, including Frankensteins, jumping jacks, pushups, mountain climbers, burpees, and some jogging. The participant was working at a standing desk and using their smartphone at their home. The participant also walked to their car and started to drive to complete some errands. The later data in the car had errors so it was trimmed to only include the participant reversing out of their parking spot.
W1	Day 1	1707956	1.98	The participant pushed and pulled carts, looked at and wrote on a clipboard or a touchpad, placed items on shelves, and bent down to objects low to the ground.
W2	Day 1	506650	0.59	The participant pushed/pulled carts, bent over to objects low to the floor and knelt on the ground to pull something once.
	Day 2	1159655	1.35	The participant operated a small touchscreen, spent some time interacting with something overhead, leaned far forward for something and bent over in other postures, as well. They also poured some material, pulled and pushed a cart, sat down for a small time, and operated machinery. The participant also operated a dolly, including putting an object onto it.
	Day 3	715716	0.83	The participant walked around while pushing an object similar to a wheelbarrow. They also pushed and pulled carts. They did some overhead lifting of materials and low-height object manipulation with various bending postures. The participant carried objects on their shoulders and spent some time looking upwards at something.
W3	Day 1	1709720	1.97	The participant sorted materials and placed objects in containers. They carried objects around. The participant also did low-height object manipulation with various bending postures. They also pushed and pulled carts.
W4	Day 1	1646669	1.9	The participant did low-height object manipulation, including squatting. They operated machinery while standing up (possibly a stand-up forklift), pushed and pulled carts, and spent a lot of time sorting bins and other containers.

## Appendix B. Histograms for the Special Test Sets

Histograms of the special test sets are shown in Figure A1 and Figure A2. Since the special test set contains less data, the distributions are not as clear as the histograms in Figure 5 and Figure 6.
